# A comparison of different population-level summary measures for randomised trials with time-to-event outcomes, with a focus on non-inferiority trials

**DOI:** 10.1177/17407745231181907

**Published:** 2023-06-20

**Authors:** Matteo Quartagno, Tim P Morris, Duncan C Gilbert, Ruth E Langley, Matthew G Nankivell, Mahesh KB Parmar, Ian R White

**Affiliations:** MRC Clinical Trials Unit, Institute of Clinical Trials and Methodology, University College London, London, UK

**Keywords:** Non-inferiority, estimands, population-level summary measures, hazard ratio, restricted mean survival time, difference in survival

## Abstract

**Background:**

The population-level summary measure is a key component of the estimand for clinical trials with time-to-event outcomes. This is particularly the case for non-inferiority trials, because different summary measures imply different null hypotheses. Most trials are designed using the hazard ratio as summary measure, but recent studies suggested that the difference in restricted mean survival time might be more powerful, at least in certain situations. In a recent letter, we conjectured that differences between summary measures can be explained using the concept of the non-inferiority frontier and that for a fair simulation comparison of summary measures, the same analysis methods, making the same assumptions, should be used to estimate different summary measures. The aim of this article is to make such a comparison between three commonly used summary measures: hazard ratio, difference in restricted mean survival time and difference in survival at a fixed time point. In addition, we aim to investigate the impact of using an analysis method that assumes proportional hazards on the operating characteristics of a trial designed with any of the three summary measures.

**Methods:**

We conduct a simulation study in the proportional hazards setting. We estimate difference in restricted mean survival time and difference in survival non-parametrically, without assuming proportional hazards. We also estimate all three measures parametrically, using flexible survival regression, under the proportional hazards assumption.

**Results:**

Comparing the hazard ratio assuming proportional hazards with the other summary measures not assuming proportional hazards, relative performance varies substantially depending on the specific scenario. Fixing the summary measure, assuming proportional hazards always leads to substantial power gains compared to using non-parametric methods. Fixing the modelling approach to flexible parametric regression assuming proportional hazards, difference in restricted mean survival time is most often the most powerful summary measure among those considered.

**Conclusion:**

When the hazards are likely to be approximately proportional, reflecting this in the analysis can lead to large gains in power for difference in restricted mean survival time and difference in survival. The choice of summary measure for a non-inferiority trial with time-to-event outcomes should be made on clinical grounds; when any of the three summary measures discussed here is equally justifiable, difference in restricted mean survival time is most often associated with the most powerful test, on the condition that it is estimated under proportional hazards.

## Background

Randomised clinical trials are often designed using a time-to-event variable as primary outcome, especially in disease areas (e.g. cancer)^1^ where interest is mainly in estimating the effect of treatment on survival. Using a time-to-event outcome naturally allows us to account for censoring^2^ and is more efficient than using simple binary outcomes.^3^

When designing a trial, it is important to carefully define the estimand(s) of interest. In the framework recently proposed by ICH,^4^ one of the building blocks in the definition of an estimand is the *population-level summary measure* used to describe results. For binary outcomes, commonly used summary measures include the risk difference (absolute), risk ratio and odds ratio (both relative).^5^ By contrast, for time-to-event outcomes, the vast majority of clinical trials are designed using the hazard ratio (HR) (relative) as the summary measure.^1^

The HR is defined as the ratio of hazard rates between the treatment and control arms. Its interpretation is clear under the assumption that the hazards in the two groups are proportional over time. However, this assumption is often not reasonable, for example when the hazards in two groups only differ at early, or later, stages of follow-up.

When interest is in an absolute contrast between the arms or when the proportional hazards assumption does not hold, alternative summary measures might be preferable. Promising summary measures include difference in restricted mean survival time (DRMST),^6^ defined as the difference in the mean survival times in the two groups up to a fixed time horizon τ, or the difference in survival proportion at time τ (DS).

In non-inferiority trials, rather than testing whether an active treatment is better than a control one, we aim to show that it is not worse by a certain amount (known as the non-inferiority margin) or more; given ancillary advantages of this active treatment, this is then considered enough to recommend its use. The margin is defined on the scale of the chosen summary measure.

In superiority trials, if assuming proportional hazards, the null hypotheses for different summary measures are the same across the event rate range, so choice of summary measure does not affect power. However, this is not true for non-inferiority trials. As a consequence, a test based on DRMST is often more powerful for the same sample size than one based on the HR. Uno et al.^7^ suggested this might always be the case, providing examples, but without matching the margins for different summary measures, that is, without ensuring the smallest non-tolerable event risk in the research arm was the same between different summary measures in the comparison. Weir and Trinquart^8^ subsequently matched the margins, showing similar results but finding some situations where HRs might be preferable. Freidlin et al.^9^ later investigated situations where a test based on the HR is more powerful. In Quartagno et al.,^10^ we explained these results using the concept of *Non-Inferiority frontiers*,^11^ that is, curves that show the non-inferiority regions defined by the null when plotting research versus control event rates, rather than focusing on the point margin only. We hypothesised that, because of the different null hypotheses, a test of non-inferiority based on DRMST would always be at least as powerful as a test based on HR, provided we estimate the two summary measures using the same model, and hence under identical assumptions. That is, when the HR *appears* to be more powerful than DRMST, the comparison is confounded by the two summary measures being based on different models used for estimation.

The aim of this article is twofold: first, we introduce and evaluate a method of estimating DRMST and DS under the proportional hazards assumption using flexible parametric survival models, comparing the power of this estimation method to that of standard non-parametric methods. Second, we test through simulation the above hypothesis about the superior power of DRMST, by estimating different summary measures under the same flexible parametric survival model and comparing with HR in terms of power, type I error and interpretation of results. We include simulations for superiority trials to show that power gains are unique to the non-inferiority setting.

## Methods

Suppose we wish to design a trial to test whether treatment A is superior/non-inferior to control C in terms of a time-to-event primary outcome. This might be, for example, time to death, or time to disease progression.

We define *S_C_*(*t*) as the probability that an individual in the control group survives up to time *t*, and *S_A_*(*t*) the corresponding probability for an individual in the research treatment arm. Similarly, we define *h_C_*(*t*) and *h_A_*(*t*) as the hazard rates at time t for an individual in the control and research arms respectively.

### Population-level summary measures

What population-level summary measure could we use to compare the two arms? Here we describe three options, and later we explain how these can be estimated.

#### HR

This is the most common measure used in trials. It is defined as the ratio of the two hazard rates

$$\mathrm{HR}=\frac{h_{A}(t)}{h_{C}(t)}$$

This is easily interpretable under the assumption that the two hazards are proportional, and hence that the chance of experiencing an event in the research arm at a specific time is always HR times that in the control arm, whatever the time. The interpretation under non-proportional hazards is less straightforward and generally requires some definition of average HR.^12,13^

The HR is an ideal summary measure when (a) we are interested in a relative difference measure and (b) the hazards are likely to be approximately proportional. When either condition does not hold, other summary measures may be preferable.

#### DRMST

This is defined as the expected difference in survival time between the research and control groups, ignoring survival after a certain time τ. Algebraically, this is the difference between the integrals of the survival functions for the two groups within a fixed time horizon τ

$$\mathrm{DRMST}(\tau )=\int_{0}^{\tau }S_{A}\left(t\right)\mathrm{dt}-\int_{0}^{\tau }S_{C}\left(t\right)\mathrm{dt}$$

It corresponds to the difference between the areas under the two survival curves. One advantage of DRMST is that its interpretation does not rely on the proportional hazards assumption. Another one is that it accounts for survival at each time point within the selected interval [0, τ], rather than just focusing on survival at time *τ*. However, this can also be a disadvantage in situations where interest actually lies in survival at time *τ* only and early differences are not considered important, or conversely whenever any difference after *τ* would still be relevant. Furthermore, the choice of τ (required) is an awkward complication compared with HR.

DRMST is a measure of absolute difference between the arms; it can be estimated in various ways, and this might be using a proportional hazards model, but does not lose its interpretation in the absence of proportional hazards.

#### DS

A common critique of DRMST is that it places too much weight on early differences in survival. In certain disease areas, only the final survival probability matters. An alternative summary measure is the difference in the survival proportions at a specified time τ

$$\mathrm{DS}(\tau )=S_{A}\left(\tau \right)-S_{C}\left(\tau \right)$$

DS is an absolute summary measure, relevant in the presence or absence of proportional hazards.

DS is a good summary measure when interest lies only in survival by a certain time point, so that whether events happened earlier or later does not matter as long as they happened before that time point. As with DRMST, this implies that we are not interested in what happens after time τ .

### Estimation methods

#### Non-parametric methods

The most famous non-parametric method is the Kaplan–Meier method for the estimation of the survival curves.^14^ This can be used to estimate both DRMST and DS. In the absence of censoring, DS could also be estimated by methods that do not estimate the whole survival curve, but only dichotomise survival at the pre-defined time point.

While non-parametric methods for the estimation of HRs have been developed,^15^ they are not commonly used in practice.^16^

#### Semi-parametric methods

These, and specifically the Cox proportional hazards model, are by far the most used in clinical trials. They assume a parametric form for the covariate effect, but leave the baseline hazard distribution unspecified. While this is an advantage when the goal is to estimate the HR, it is not possible to estimate DRMST and DS with associated confidence intervals without resorting to bootstrap.

#### Fully parametric methods

These methods assume a parametric model for both parts of the hazard function. Common options include exponential and Weibull functions for the baseline hazard $h_{0}(t)$ . However, in the absence of prior knowledge about the likely distribution of the baseline hazards, flexible methods based on estimation with spline functions^17^ might be preferable. With flexible parametric survival models, it is straightforward to estimate HR, but even the estimation of DRMST and DS under proportional hazards with the associated confidence intervals only requires simple post-processing of the model parameter estimates. See the Supplementary material for details.

#### Choice of methods

In clinical trials, most often non-parametric methods are used to estimate DRMST and DS, while HR is almost always estimated using semi-parametric methods (i.e. the Cox proportional-hazards model). In this article, though, we additionally use fully parametric models to estimate all summary measures, in order to make a fairer comparison between them by using the same model. While using flexible fully parametric models to estimate HR is not expected to lead to noticeable differences compared to Cox models,^18^ we hypothesise that using the same models to estimate DRMST and DS under proportional hazards could lead to substantial power gains compared to standard non-parametric methods.

### Non-inferiority trials

The choice of population-level summary measure is particularly important in non-inferiority trials. In such trials, the non-inferiority margin has to be chosen, and this is expressed as a value of the population-level summary measure of treatment effect that would be considered non-tolerable even in the presence of secondary advantages of the research treatment.

The choice of summary measure is more important than in superiority trials because different measures imply different null hypotheses^10^ (see the Supplementary material for further details).

Despite this issue, it is possible to match margins between different summary measures by using the expected values, though the null hypotheses remain different.

### Simulation study

We now describe a simulation study to investigate our research questions.^19^

#### Aim

The aims of this simulation study are as follows:

1. To compare different summary measures when analysing under the proportional hazards assumption.
2. To compare the impact of assuming proportional hazards in the estimation method on the properties of the different summary measures.

#### Data generating mechanisms

We use the data generating mechanisms described by Freidlin et al.^9^ Participants are recruited uniformly over 3 years and are followed until the end of 6 years from start of trial recruitment. The baseline hazard is constant, so that survival times follow an exponential distribution, and the scale parameter is chosen to lead to 3-year survival of 20%, 60% or 90%. Data are generated from the null and alternative hypothesis of both superiority trials with varying effect magnitude and non-inferiority trials with varying margins. Sample sizes are chosen to lead to commonly used power levels, that is, greater than 80%, when designing the trial using HR. Precise parameter values are listed in Tables 1 and 2.

**Table 1.** Comparison of type I error rates for tests based on different summary measures in proportional hazards non-inferiority scenarios.

Scenario	Design parameters					Type I error (%)							
	S(3) control (%)	Sample size	Non-inferiority margin			Non-parametric		Flexible parametric under PH using data to 3 years			Flexible parametric under PH using all data		
			HR	DRMST(3)	DS(3) (%)	DRMST(3)	DS(3)	HR	DRMST(3)	DS(3)	HR	DRMST(3)	DS(3)
13	90	250	2	0.143	9	2.84	2.04	2.63	3.60	2.69	2.55	4.02	2.78
14	90	450	1.75	0.108	7	2.95	2.11	2.73	3.32	2.77	2.59	3.58	2.83
15	90	1000	1.5	0.073	5	2.43	2.03	2.38	2.61	2.46	2.63	3.12	2.79
16	90	3750	1.25	0.037	2	2.53	2.30	2.53	2.58	2.52	2.45	2.84	2.68
17	60	75	2	0.469	24	2.39	1.76	2.49	2.72	2.32	2.36	2.92	2.23
18	60	125	1.75	0.366	19	2.51	2.01	2.72	3.00	2.64	2.61	2.81	2.41
19	60	250	1.5	0.253	14	2.51	2.13	2.84	2.75	2.60	2.65	2.65	2.52
20	60	1000	1.25	0.132	7	2.51	2.28	2.47	2.47	2.48	2.62	2.56	2.53
21	20	50	2	0.596	16	2.45	1.98	2.31	2.36	5.44	2.28	2.30	5.13
22	20	75	1.75	0.490	14	2.49	1.97	2.50	2.44	4.15	2.56	2.50	3.93
23	20	150	1.5	0.360	11	2.65	1.88	2.61	2.58	3.40	2.53	2.58	3.13
24	20	450	1.25	0.199	7	2.62	2.02	2.60	2.61	2.71	2.51	2.50	2.64

DRMST(3): difference in restricted mean survival time to 3 years, DS(3): difference in 3-year survival probability, HR: hazard ratio, PH: proportional hazards, S(3): 3-year survival.

Monte Carlo standard errors can be computed as $\sqrt{p(1-p)/n}$ , and are generally in the order of 0.15%. Entries in red indicate scenarios for which type 1 error rate is greater than 3%.

**Table 2.** Comparison of power for tests based on different summary measures in proportional hazards non-inferiority scenarios.

Scenario	Design parameters					Power (%)							
	S(3) control (%)	Sample size	Non-inferiority margins			Non-parametric		Flexible parametric under PH using data to 3 years			Flexible parametric under PH using all data		
			HR	DRMST(3)	DS(3) (%)	DRMST(3)	DS(3)	HR	DRMST(3)	DS(3)	HR	DRMST(3)	DS(3)
13	90	250	2	0.143	9	85.0	89.6	69.0	93.7^a^	91.9	83.5	98.2^a^	97.8
14	90	450	1.75	0.108	7	86.1	91.0	75.1	93.7^a^	92.5	88.9	98.5^a^	98.2
15	90	1000	1.5	0.073	5	85.6	92.2	81.3	93.7	93.2	92.8	98.5^a^	98.3
16	90	3750	1.25	0.037	2	83.8	91.3	85.8	92.3	91.9	95.7	98.3	98.2
17	60	75	2	0.469	24	84.8	82.1	75.6	92.5	85.9	85.9	97.2	94.0
18	60	125	1.75	0.366	19	84.2	82.7	78.7	91.6^a^	86.8	89.1	97.0	94.4
19	60	250	1.5	0.253	14	83.5	85.2	81.5	90.6	87.6	91.0	96.6	95.0
20	60	1000	1.25	0.132	7	86.7	90.3	88.8	92.8	91.8	95.4	97.4	96.9
21	20	50	2	0.596	16	81.9	48.7	87.0	86.0	62.0^a^	90.2	89.4	66.6^a^
22	20	75	1.75	0.490	14	82.0	54.2	85.8	85.7	66.9^a^	89.7	89.5	71.5^a^
23	20	150	1.5	0.360	11	84.8	61.6	88.1	88.4	75.7^a^	91.5	91.5	80.5^a^
24	20	450	1.25	0.199	7	82.0	67.7	85.0	85.4	79.3	89.6	89.9	84.2

S(3): 3-year survival probability; PH: proportional hazards; HR: hazard ratio; DRMST(3): difference in restricted mean survival time to 3 years; DS(3): difference in 3-year survival probability.

Monte Carlo standard errors can be computed as $\sqrt{p(1-p)/n}$ , and are generally in the order of 0.3–0.4%.

a Scenario where type I error was >3%.

For all scenarios, we simulate 10,000 repetitions, which should give a Monte Carlo standard error around 0.15% for type I error and below 0.5% for power across scenarios.

#### Estimands

We assume that all patients are followed up to the end of 6 years of the study, and adherence to randomised group is perfect. Hence, our comparison focuses on the population-level summary measure. We compare HR, DRMST at 3 years and DS at 3 years.

#### Methods

We estimate all summary measures after fitting a flexible parametric survival model under proportional hazards, with two internal spline knots placed at the 33% and 67% quantiles of the uncensored survival times. We fit this flexible parametric model on the time-since-randomisation scale, either using all available information or censoring after 3 years. These two options correspond to the ‘staggered’ and ‘non-staggered’ scenarios in Freidlin et al.^9^ and they are both included to show how part of the expected power gain with flexible parametric models comes from the fact that, assuming proportional hazards, data after 3 years would inform estimation through the proportional hazards assumption. In addition, we estimate DRMST and DS non-parametrically: for the former, we use the Kaplan–Meier method with the Greenwood variance formula^20^; for the latter, we compute the difference in proportions at 3 years and a Wald confidence interval. Note that non-parametric results are unaffected by inclusion of data beyond 3 years, because of the lack of distributional assumptions.

#### Performance measures

We focus on power and type I error.

#### Implementation

For a handful of repetitions, flexible parametric models experienced convergence issues. Because our main interest is in comparing summary measures, rather than methods, we decided to simply discard and replace such repetitions. Of note, since this happened in seven repetitions out of approximately 300,000 across scenarios, it is unlikely to have had any impact even in the comparison of different methods.

## Results

### Simulation study

**Figure 1.**
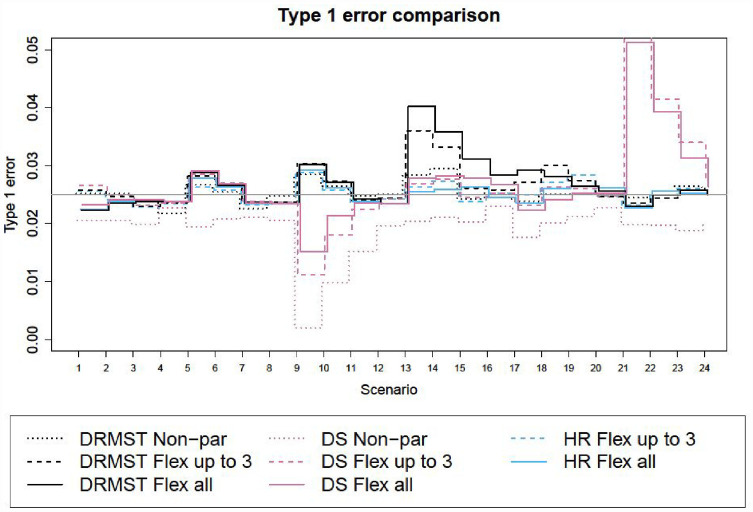
Type I error for different methods across scenarios. The nominal level is 2.5%. DRMST: difference in restricted mean survival time; DS: difference in 3-year survival probability; HR: hazard ratio; Non-par: non-parametric analysis method; Flex: flexible fully parametric survival model under proportional hazards; all: using all data; up to 3: using data to 3 years.

**Figure 2.**
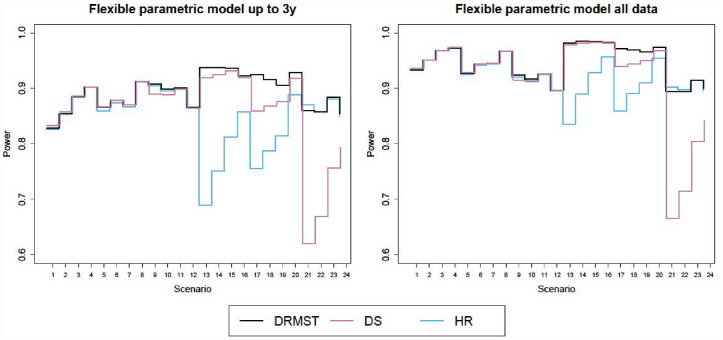
Power of different flexible parametric models under proportional hazards across scenarios, either using data up to 3 years only (left panel) or all the data (right panel). DRMST: difference in restricted mean survival time; DS: difference in 3-year survival probability; HR: hazard ratio.

Figures 1 and 2 show the results in terms of type I error and power respectively, for superiority trials and non-inferiority trials. Corresponding numerical results are in Tables a and b in the additional online material for superiority trials (scenarios 1–12) and in Tables 1 and 2 for non-inferiority trials (scenarios 13–24).

#### Type I error evaluation

Type I error is controlled in most scenarios and methods, although there are a few scenarios where the delta method approximation of the standard error used for DRMST and DS leads to slight inflation (Figure 1). This is most likely due to limited sample size, as the inflation disappears with larger samples. We propose possible solutions to this in section ‘Discussion’.

#### Comparison of summary measures

When using standard methods (i.e. non-parametric models for DRMST and DS and proportional hazards models for HR), the relative performance of DRMST and HR varies with scenarios as described in Freidlin et al.,^9^ while DRMST is more powerful than DS only for 20% survival (Table 2, scenarios 21–24). However, if we compare DRMST, DS and HR under the same assumptions, that is, estimating them with flexible proportional hazards parametric models using either data up to 3 years or all the available data (Figure 2), as expected from theory, power is always similar for superiority trials (scenarios 1–12). In non-inferiority trials (scenarios 13–24), DRMST fares much better in some scenarios, slightly better in some others, and marginally worse in scenario 21.

When comparing DS with DRMST and HR, conclusions depend on the specific scenario, but DRMST is always at least as powerful as DS, and is more powerful in several non-inferiority scenarios (Figure 2).

#### Impact of proportional hazards assumption

Estimating DRMST and DS using flexible parametric models under the proportional hazards assumption always leads to gains in power compared to non-parametric estimation. Using all the data, rather than censoring at 3 years, leads to another increase in power, even for estimands defined at 3 years, like DRMST and DS. This is because, under the proportional hazards assumption, even later events can help learn more about earlier time points.

### The PATCH clinical trial

PATCH (ISRCTN70406718) is a non-inferiority randomised controlled trial comparing two different strategies of androgen suppression in men with prostate cancer.^21^ Standard therapies (Luteinising hormone-releasing hormone analogue injections) are effective at lowering testosterone and controlling cancer, but can cause serious long-term side effects, particularly osteoporosis. Transdermal oestradiol patches are an alternative approach with potentially a better side effect profile.^22^

Patients with locally advanced but non-metastatic disease and those with metastatic disease were originally evaluated as a single group within the trial. However, with evolving standards of care, expected outcomes for these different groups of patients have diverged. Consequently, the trial was recently divided into two separate non-inferiority trials:

1. Non-metastatic disease trial: The primary outcome measure is metastasis-free survival, and the trial is 85% powered to detect non-inferiority within a non-inferiority margin on the HR scale of 1.27, with a 5% one-sided significance level. This assumes that 3-year metastasis-free survival would be 83% in the control arm; it requires around 510 events, out of a target sample size of 1345 patients.
2. Metastatic disease trial: The primary outcome measure is overall survival, and the trial is 80% powered to detect non-inferiority within a margin of 1.19 on the HR scale, with a 5% one-sided significance level. This is under the assumption that 3-year overall survival in the control arm will be around 66%; it requires around 822 deaths, out of a target sample size 1,500.

Both trials have allocation ratio 1.08:1, because an original phase II design with 2:1 allocation ratio was expanded seamlessly to a larger phase III trial with 1:1 allocation ratio.

The HR margin was originally justified based on an absolute DS; for example, for non-metastatic disease the margin was set as a DS at 3 years of 4 percentage points and the corresponding HR margin was back-calculated from the control arm rate. The corresponding DRMST margin with τ= 3 years would have been equally justifiable. To match the margins used for the HR, we calculated a DRMST margin of 0.0658 and a DS margin of 4.00 percentage points for non-metastatic patients, and a DRMST margin of 0.0879 and a DS margin of 5.00 percentage points for metastatic patients. We generated data similar to the simulation study, but using the trial design parameters, and compared power for both cohorts to detect non-inferiority using each of the three summary measures, again using parametric and non-parametric methods.

Table 3 shows the results. Assuming proportional hazards leads to greater power and should therefore be preferred, since hazards are expected to be approximately proportional in the PATCH trial. In terms of summary measures, DRMST seems preferable, leading to power 6 and 4.5 percentage points higher in the two cohorts.

**Table 3.** Power of the 2 cohorts in the PATCH trial using different summary measures to define the margin, and analysing the trial either non-parametrically or parametrically using flexible parametric survival models under proportional hazards.

Scenario	Design parameters					Power (%)				
	S(3) control (%)	Sample size (total)	Non-inferiority margins			Non-parametric		Flexible parametric under PH using all data		
			HR	DRMST(3)	DS(3) (%)	DRMST(3)	DS(3)	HR	DRMST(3)	DS(3)
13	83	1345	1.27	0.0658	4	55.4	60.6	85.6	91.6	89.8
14	66	1500	1.19	0.0879	5	57.6	62.9	79.4	83.9	82.6

PATCH: prostate adenocarcinoma transcutaneous hormone; S(3): 3-year survival probability; PH: proportional hazards; HR: hazard ratio; DRMST(3): difference in restricted mean survival time to 3 years; DS(3): difference in 3-year survival probability.

## Discussion

In this article, we have compared three population-level summary measures for clinical trials with time-to-event outcomes when analysed under a correct proportional hazards assumption.

When the hazards are proportional, analysing the data with a model that reflects their proportionality leads to big improvement in power, compared to the standard approach of estimating DRMST and DS non-parametrically.

When using standard methods for estimation, that is, Cox models for HR and non-parametric methods for DRMST and DS, the relative performance of summary measures varies substantially depending on the design parameters, as previously observed.^9^ However, if using the methods proposed here, which base the estimation of all summary measures on fitting the same flexible parametric model under proportional hazards, we can conclude that:

- For superiority trials, the null hypotheses correspond for all three summary measures, and therefore testing on any summary measure leads to similar power levels.
- For non-inferiority trials, DRMST assuming proportional hazards is almost always at least as powerful as – and often more powerful than – HR, except in rare cases, for example, in scenario 21, where survival probability at τ approaches zero and the non-inferiority margin is large. The relative performance for DS depends on design parameters.

Thus, some of the apparent differences between HR and DRMST observed in Freidlin et al.^9^ were due to the different modelling assumptions of the estimators. For readers interested in the reasons for these results, the Supplementary material includes a short discussion using our recently proposed graphical tool, the *non-inferiority frontier*.^10,11^

In this article, we have assumed proportional hazards throughout. Non-proportionality of hazards may potentially have huge impact in terms of power and/or type I error. Therefore, future work will investigate this and possibly derive specific methods to address it. For example, weighting could be used to get an unbiased estimate of the average HR^23^ under non-proportionality of the hazards,^13^ and the same approach could be investigated in the future for other summary measures. At present though, for DRMST and DS, non-parametric methods should be preferred when the hazards are not expected to be proportional.

The delta method that we used for estimating standard errors for DRMST and DS from the flexible parametric model relies on asymptotic arguments and can hence be poorly calibrated with smaller sample sizes. Preliminary simulations suggest that a non-parametric bootstrap strategy might lead to better control of type I error; however, while this might be preferable for a single analysis, it was too computationally intensive for this simulation study. Further, the non-parametric bootstrap itself relies on asymptotic arguments so would need to be studied further. Importantly, while type 1 error might be wrongly controlled in some of our scenarios, this did not appear to have the potential to have any impact on the conclusions of our study in terms of power.

While most trials that target the HR use Cox models, we used flexible parametric models here for comparability of models across summary measures, since we could not find an analytic way to estimate standard errors around DRMST based on Cox model estimates without resorting to bootstrap. Nevertheless, results of separate simulations (not shown) confirm^18^ that differences between Cox and flexible parametric models are minimal. Relatedly, since both Cox and flexible parametric methods can handle various types of baseline hazard function, while data were always generated from exponential models in the proportional hazards scenarios of our simulations, we expect results would not differ under different baseline hazard distributions.

### Recommendations

1. The choice between different summary measures should be driven first by clinical considerations. Power considerations should only determine choice of summary measure among summary measures that are acceptable clinically.
2. All assumptions and analysis methods being equal, it is advisable to choose the most powerful summary measure. This is often the DRMST, provided it is estimated under proportional hazards.
3. Whenever the proportional hazards assumption is likely to hold, reflecting it in the analysis is recommended in order to maximise power.

## Conclusion

Both the choice of summary measure and analysis method are very important for clinical trials designed with time-to-event outcomes, particularly for non-inferiority trials. Simply relying on the HR estimated with a Cox model as a default should be discouraged. Clinical considerations should come first to choose a meaningful summary measure. If clinical considerations are equal and the hazards are likely to be proportional, then DRMST estimated under proportional hazards seems preferable, since it nearly always has power better or the same as HR or DS.

## Supplemental Material

sj-docx-1-ctj-10.1177_17407745231181907 – Supplemental material for A comparison of different population-level summary measures for randomised trials with time-to-event outcomes, with a focus on non-inferiority trials Supplemental material, sj-docx-1-ctj-10.1177_17407745231181907 for A comparison of different population-level summary measures for randomised trials with time-to-event outcomes, with a focus on non-inferiority trials by Matteo Quartagno, Tim P Morris, Duncan C Gilbert, Ruth E Langley, Matthew G Nankivell, Mahesh KB Parmar and Ian R White in Clinical Trials
