# Supplementary material for: A comparison of different population-level summary measures for randomised trials with time-to-event outcomes, with a focus on non-inferiority trials
Source: Clin Trials. Author manuscript; Available in PMC 2023 Dec 1. (PMC7615295; doi:10.1177/17407745231181907)
Supplement: Supplementary material [file EMS177448-supplement-Supplementary_material.docx]

| Scenario | Design parameters | | | | | Type I error | | | | | | | | |
| --- | --- | --- | --- | --- | --- | --- | --- | --- | --- | --- | --- | --- | --- | --- |
|  | S(3) control | Sample size | Delta (alternative hypothesis) | | | Non-Parametric | | Flexible parametric under PH using data to 3 years | | | Flexible parametric under PH using all data | | | |
|  |  |  | HR | DRMST(3) | DS(3) | DRMST(3) | DS(3) | HR | DRMST(3) | DS(3) | | HR | DRMST(3) | DS(3) |
| 13 | 90% | 250 | 2 | 0.143 | 9% | 2.52% | 2.05% | 2.56% | 2.58% | 2.66% | | 2.25% | 2.23% | 2.33% |
| 14 | 90% | 450 | 1.75 | 0.108 | 7% | 2.52% | 2.05% | 2.41% | 2.47% | 2.50% | | 2.38% | 2.36% | 2.41% |
| 15 | 90% | 1000 | 1.5 | 0.073 | 5% | 2.40% | 1.99% | 2.29% | 2.30% | 2.31% | | 2.41% | 2.39% | 2.41% |
| 16 | 90% | 3750 | 1.25 | 0.037 | 2% | 2.18% | 2.27% | 2.35% | 2.36% | 2.36% | | 2.38% | 2.38% | 2.38% |
| 17 | 60% | 75 | 2 | 0.469 | 24% | 2.67% | 1.94% | 2.63% | 2.82% | 2.85% | | 2.79% | 2.88% | 2.91% |
| 18 | 60% | 125 | 1.75 | 0.366 | 19% | 2.55% | 2.08% | 2.58% | 2.67% | 2.70% | | 2.63% | 2.66% | 2.69% |
| 19 | 60% | 250 | 1.5 | 0.253 | 14% | 2.26% | 2.11% | 2.33% | 2.37% | 2.39% | | 2.34% | 2.36% | 2.36% |
| 20 | 60% | 1000 | 1.25 | 0.132 | 7% | 2.48% | 2.06% | 2.36% | 2.37% | 2.37% | | 2.35% | 2.35% | 2.35% |
| 21 | 20% | 50 | 2 | 0.596 | 16% | 2.85% | 0.20% | 2.88% | 3.04% | 1.12% | | 2.92% | 3.02% | 1.51% |
| 22 | 20% | 75 | 1.75 | 0.490 | 14% | 2.65% | 0.98% | 2.58% | 2.73% | 1.80% | | 2.62% | 2.71% | 2.14% |
| 23 | 20% | 150 | 1.5 | 0.360 | 11% | 2.48% | 1.52% | 2.37% | 2.40% | 2.25% | | 2.39% | 2.42% | 2.36% |
| 24 | 20% | 450 | 1.25 | 0.199 | 7% | 2.51% | 1.96% | 2.43% | 2.44% | 2.43% | | 2.35% | 2.35% | 2.35% |

Table a: Comparison of type I error rates for tests based on different summary measures for proportional hazards superiority scenarios. Monte Carlo standard error can be computed as $\sqrt{\frac{\boldsymbol{p(1-p)}}{\boldsymbol{n}}}$, and are generally in the order of 0.15%.

|  | Design parameters | | | | | Power | | | | | | | | |
| --- | --- | --- | --- | --- | --- | --- | --- | --- | --- | --- | --- | --- | --- | --- |
|  | S(3) control | Sample size | Delta (alternative hypothesis) | | | Non-Parametric | | | Flexible parametric under PH using data to 3 years | | | Flexible parametric under PH using all data | | |
| Scenario |  |  | HR | DRMST(3) | DS(3) | | DRMST(3) | DS(3) | HR | DRMST(3) | DS(3) | HR | DRMST(3) | DS(3) |
| 13 | 90% | 250 | 2 | 0.143 | 9% | | 72.4% | 79.6% | 82.6% | 82.9% | 83.3% | 93.5% | 93.3% | 93.7% |
| 14 | 90% | 450 | 1.75 | 0.108 | 7% | | 76.0% | 83.3% | 85.3% | 85.5% | 85.7% | 95.1% | 95.1% | 95.2% |
| 15 | 90% | 1000 | 1.5 | 0.073 | 5% | | 79.0% | 86.9% | 88.4% | 88.5% | 88.6% | 96.8% | 96.8% | 96.8% |
| 16 | 90% | 3750 | 1.25 | 0.037 | 2% | | 80.6% | 89.6% | 90.2% | 90.2% | 90.2% | 97.3% | 97.3% | 97.3% |
| 17 | 60% | 75 | 2 | 0.469 | 24% | | 80.3% | 82.3% | 85.9% | 86.5% | 86.6% | 92.5% | 92.7% | 92.8% |
| 18 | 60% | 125 | 1.75 | 0.366 | 19% | | 81.1% | 83.0% | 87.4% | 87.8% | 87.9% | 94.2% | 94.4% | 94.5% |
| 19 | 60% | 250 | 1.5 | 0.253 | 14% | | 79.6% | 84.6% | 86.7% | 87.0% | 87.0% | 94.4% | 94.5% | 94.6% |
| 20 | 60% | 1000 | 1.25 | 0.132 | 7% | | 84.5% | 89.6% | 91.2% | 91.2% | 91.3% | 96.7% | 96.7% | 96.7% |
| 21 | 20% | 50 | 2 | 0.596 | 16% | | 89.1% | 63.3% | 90.4% | 90.8% | 89.0% | 92.0% | 92.4% | 91.5% |
| 22 | 20% | 75 | 1.75 | 0.490 | 14% | | 87.3% | 67.0% | 89.5% | 89.9% | 88.9% | 91.4% | 91.7% | 91.3% |
| 23 | 20% | 150 | 1.5 | 0.360 | 11% | | 88.2% | 74.5% | 90.0% | 90.1% | 89.9% | 92.5% | 92.7% | 92.6% |
| 24 | 20% | 450 | 1.25 | 0.199 | 7% | | 83.5% | 73.1% | 86.4% | 86.5% | 86.5% | 89.6% | 89.7% | 89.6% |

Table b: Comparison of power for tests based on different summary measures in proportional hazards superiority scenarios. Monte Carlo standard error can be computed as $\sqrt{\frac{\boldsymbol{p(1-p)}}{\boldsymbol{n}}}$, and are generally in the order of 0.3-0.4%.

**Details of method implementation:**

**Non-parametric methods**. Non-parametric methods were already implemented in commonly used R packages. Hence, we simply used such packages.
In particular, for non-parametric implementation of the DRMST method we used function rmst2 from package survRM2 which uses the delta method to estimate DRMST from the Kaplan-Meier estimates.
For DS, we used the standard function prop.test, which tests the difference in proportions using the Newcombe 10 method. Note we were able to use this method because of the absence of censoring.

**Parametric methods.** The first step in the implementation of all parametric methods was fitting a flexible parametric survival regression model using function flexsurvspline from package flexsurv. We fitted the model either using data up to 3 years or all the available data, including treatment as the only covariate and with two internal knots. The algebraic form of the model is therefore:

$$\ln\left( H\left( t | A \right) \right)=\ln\left( H_{0}\left( t \right) \right)+\beta A=\eta_{0}+\eta_{1}z_{1}+\eta_{2}z_{2}+{4\eta}_{3}z_{3}$$

$$z_{1}=ln(t)$$

$$z_{j}={(\ln\left( t \right)-k_{j})}_{+}^{3}-\frac{k_{3}-k_{j}}{k_{3}-k_{1}}{(\ln\left( t \right)-k_{1})}_{+}^{3}-\left( 1-\frac{k_{3}-k_{j}}{k_{3}-k_{1}} \right){(\ln\left( t \right)-k_{j})}_{+}^{3}$$

Therefore, our outcome from the model is 5 parameter estimates and the associated variance-covariance matrix:

$$\left( \begin{matrix} \begin{matrix} \beta\\ \eta_{0} \\ \eta_{1} \end{matrix} \\ \eta_{2} \\ \eta_{3} \end{matrix} \right) \Omega_{e}$$

Both the difference in survival at 3 years and DRMST with tau=3 can be written as a function of these parameters:

$$DS\left( \beta,\eta_{0},\eta_{1},\eta_{2},\eta_{3} \right)=\eta_{0}+\eta_{1}z_{1}+\eta_{2}z_{2}+{4\eta}_{3}z_{3}+\beta-\eta_{0}+\eta_{1}z_{1}+\eta_{2}z_{2}+{4\eta}_{3}z_{3} t=3$$

$$DRMST\left( \beta,\eta_{0},\eta_{1},\eta_{2},\eta_{3} \right)=\int_{0}^{3} (\eta_{0}+\eta_{1}z_{1}+\eta_{2}z_{2}+{4\eta}_{3}z_{3}+\beta)dt-\int_{0}^{3} (\eta_{0}+\eta_{1}z_{1}+\eta_{2}z_{2}+{4\eta}_{3}z_{3})dt$$

According to the Delta method, their distributions can be approximated by:

$$DS\left( \beta,\eta_{0},\eta_{1},\eta_{2},\eta_{3} \right)\underset{\to}{d}N\left( DS\left( \hat{\beta},\hat{\eta_{0}},\hat{\eta_{1}},\hat{\eta_{2}},\hat{\eta_{3}} \right) ,\boldsymbol{J}_{\boldsymbol{DS}}\left( \hat{\beta},\hat{\eta_{0}},\hat{\eta_{1}},\hat{\eta_{2}},\hat{\eta_{3}} \right)\boldsymbol{\Omega}_{\boldsymbol{e}}{\boldsymbol{J}_{\boldsymbol{DS}}\left( \hat{\beta},\hat{\eta_{0}},\hat{\eta_{1}},\hat{\eta_{2}},\hat{\eta_{3}} \right)}^{T} \right)$$

$$DRMST\left( \beta,\eta_{0},\eta_{1},\eta_{2},\eta_{3} \right)\underset{\to}{d}N\left( DRMST\left( \hat{\beta},\hat{\eta_{0}},\hat{\eta_{1}},\hat{\eta_{2}},\hat{\eta_{3}} \right) ,\boldsymbol{J}_{\boldsymbol{DRMST}}\left( \hat{\beta},\hat{\eta_{0}},\hat{\eta_{1}},\hat{\eta_{2}},\hat{\eta_{3}} \right)\boldsymbol{\Omega}_{\boldsymbol{e}}{\boldsymbol{J}_{\boldsymbol{DRMST}}\left( \hat{\beta},\hat{\eta_{0}},\hat{\eta_{1}},\hat{\eta_{2}},\hat{\eta_{3}} \right)}^{T} \right)$$

Where $\boldsymbol{J}_{\boldsymbol{DS}}$ and $\boldsymbol{J}_{\boldsymbol{DRMST}}$ are the Jacobians for the two functions, i.e. the functions with the five partial derivatives against the five parameters. We estimated these numerically with the grad function in the NumDeriv R package.

**Non-inferiority Frontiers:**

The results of our simulations comparing power between different summary measures, when analysed using the same analysis method, can be explained with our recently proposed graphical tool, the *non-inferiority frontiers*, *i.e.* the curves indicating the smallest non-tolerable values of research event rate corresponding to each value of control event rate when using a specific summary measure. This is illustrated by Figure (a), which depicts the non-inferiority frontiers corresponding to different summary measures for three of the scenarios in our simulation study. In each case, the null hypothesis is that the true event rates define a point on or above the non-inferiority frontier. Because the frontiers differ, the null hypotheses differ. We can see that in the left panel (Scenario 13), with large margins and small event rates, the frontier related to the HR passes much closer to the expected point than that related to the other two summary measures, meaning that a test using HR will need larger sample size. In the central panel, with larger event risk and smaller margin (Scenario 20), the differences are modest. In the right panel (Scenario 21) the DS frontier passes the closest, with DRMST second and HR marginally further away. In general, the closer the frontier, the lower the power to reject the null hypothesis. Note that, in the same figure, the corresponding “superiority frontier” would simply be the equality line, which would be the same for all measures, implying identical null hypotheses.

Non-inferiority frontiers can be used to choose the most powerful summary measure when more than one is clinically justifiable. Function frontier.comparison.survival in the R package dani can be used to do this. This is currently available in the GitHub page of the first author (<https://github.com/Matteo21Q>) and will be soon submitted to CRAN.


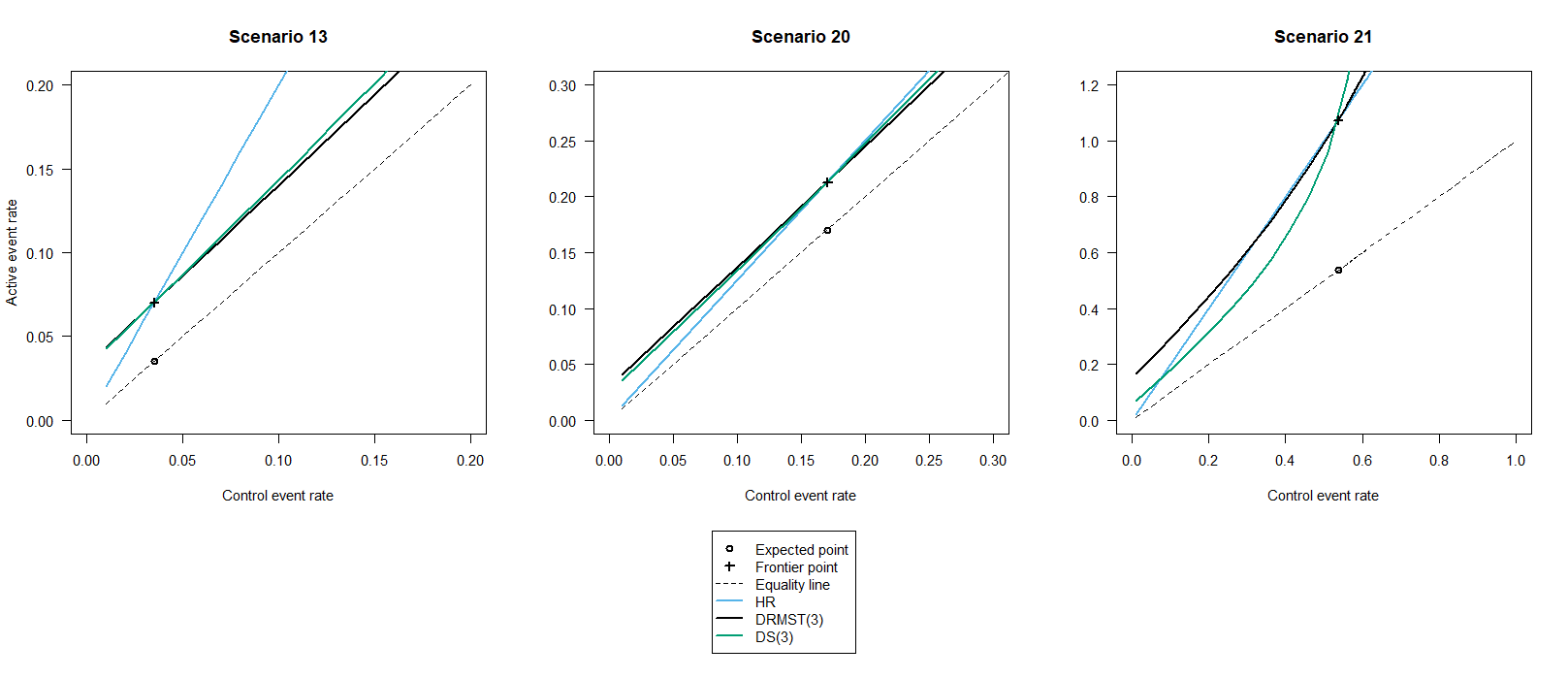


Figure a: Comparison of three Non-Inferiority frontiers based on Hazard Ratio (HR), Difference in Restricted Mean Survival Time (DRMST) and Difference in Survival probability (DS) margins. These are shown for scenarios 13, 20 and 21 of the simulation study.
